# Characterization of Patients with Poor Clinical Outcome after Adult Spinal Deformity Surgery: A Multivariate Analysis of Mean 8-Year Follow-Up Data

**DOI:** 10.3390/jcm13196000

**Published:** 2024-10-08

**Authors:** Se-Jun Park, Hyun-Jun Kim, Jin-Sung Park, Dong-Ho Kang, Minwook Kang, Kyunghun Jung, Chong-Suh Lee

**Affiliations:** 1 Department of Orthopedic Surgery, Spine Center, Samsung Medical Center, Sungkyunkwan University School of Medicine, Seoul 06351, Republic of Korea; sejunos@gmail.com (S.-J.P.); paridot@daum.net (J.-S.P.); kang9451@gmail.com (D.-H.K.); npng4eve@gmail.com (M.K.); ilucky7ik@gmail.com (K.J.); 2 Department of Orthopedic Surgery, Hanyang University Guri Hospital, Hanyang University School of Medicine, Guri-si 11923, Republic of Korea; 3 Department of Orthopedic Surgery, Haeundae Bumin Hospital, Busan 48094, Republic of Korea; csl3503@gmail.com

**Keywords:** adult spinal deformity, poor clinical outcome, risk factor, proximal junctional kyphosis, long-term follow-up, health-related quality of life

## Abstract

**Background/Objective:** Limited data exist regarding the long-term clinical outcomes and related factors after adult spinal deformity (ASD) surgery. This study aims to characterize patients who experienced poor clinical outcomes during long-term follow-up after ASD surgery. **Methods:** Patients who underwent ASD surgery with ≥5-vertebra fusion including the sacrum and ≥5-year follow-up were included. They were divided into two groups according to the Oswestry Disability Index (ODI) at the last follow-up: group P (poor outcome, ODI > 40) and group NP (non-poor outcome, ODI ≤ 40). Clinical variables, including patient factors, surgical factors, radiographic parameters, and mechanical complications (proximal junctional kyphosis [PJK] and rod fracture), were compared between the groups. **Results:** A total of 105 patients were evaluated, with a mean follow-up of 100.6 months. The mean age was 66.3 years, and 94 patients (89.5%) were women. There were 52 patients in group P and 53 patients in group NP. Univariate analysis showed that low T-score, postoperative correction relative to age-adjusted pelvic incidence-lumbar lordosis, T1 pelvic angle (TPA) at last follow-up, and PJK development were significant factors for poor clinical outcomes. Multivariate analysis identified PJK as the single independent risk factor (odds ratio [OR] = 3.957 for PJK development relative to no PJK, OR = 21.141 for revision surgery for PJK relative to no PJK). **Conclusions:** PJK development was the single independent factor affecting poor clinical outcomes in long-term follow-up. Therefore, PJK prevention appears crucial for achieving long-term success after ASD surgery.

## 1. Introduction

Adult spinal deformity (ASD) is a disabling condition that causes significant pain and disability, resulting in a marked decline in the patient’s health-related quality of life (HRQOL) [1]. Since sagittal imbalance leads to poor HRQOL [2,3,4,5], proper spinopelvic malalignment correction has been prioritized as a crucial surgical goal. Previous ASD-related studies have uniformly emphasized the importance of correcting spinopelvic malalignment for the success of surgery [2,3,4,5,6,7,8]. However, this evidence is insufficient to determine whether the role of optimal sagittal alignment in clinical outcomes remains valid during long-term follow-up or if other factors influence clinical outcomes over time.

Along with adequate postoperative sagittal alignment, other factors that might negatively affect the clinical outcomes should be considered when assessing the long-term outcomes after ASD surgery. First, whether postoperatively restored sagittal alignment will be maintained over long-term follow-up should be considered, as many patients experience some degree of correction loss over time [9,10,11]. The loss of correction can occur within the fusion segments and unfused thoracic spine, both of which may deteriorate the global sagittal balance. Second, mechanical complications such as proximal junctional kyphosis (PJK) and rod fracture can arise, negatively impacting final clinical outcomes [12,13,14]. It is known that the development of mechanical complications is associated with postoperative sagittal alignment status [15,16]. However, the adequate correction of sagittal alignment cannot completely prevent mechanical failure [17]. Moreover, the incidence of these mechanical complications and the risk of related revision surgery continuously increase over time [18,19].

For assessing the long-term clinical outcome after ASD surgery, it is necessary to comprehensively consider various clinical parameters, including demographics, immediate postoperative and final radiographic findings, and mechanical complications, because these factors may be closely related and can affect the final clinical outcome. In the literature, data regarding the long-term clinical outcomes are limited. Furthermore, it has not been clearly established which factors are most responsible for poor clinical outcomes after ASD surgery. Therefore, this study aims to characterize patients who experience poor clinical outcomes using multivariate analysis of mean 8-year follow-up data.

## 2. Materials and Methods

This study received approval from Samsung Medical Center’s institutional review board (IRB No. SMC 2024-07-144). Given its retrospective design, the requirement for informed consent was waived.

### 2.1. Study Cohort

This research involved a retrospective case series utilizing data extracted from our hospital’s prospective ASD database. The study population comprised consecutive patients who underwent surgery for degenerative ASD (i.e., degenerative flatback [DFB] or degenerative lumbar scoliosis [DLS]) during 2010–2019. Patients were included based on the following criteria: ASD was defined by radiographic measurements, including a C7 sagittal vertical axis (C7SVA) of 50 mm or greater, a pelvic incidence (PI)-lumbar lordosis (LL) mismatch of 10° or more, a pelvic tilt (PT) of 25° or more, or a lumbar coronal Cobb angle of 20° or greater. In addition, the fusion involved at least five vertebral levels, all of which included the sacrum to minimize bias related to fusion length. Patients were also required to have at least five years of complete radiographic and HRQOL data. Exclusion criteria were incomplete radiographs, failure to complete the HRQOL questionnaire at the final follow-up, prior thoracic or lumbar fusion, or the presence of syndromic, neuromuscular, inflammatory, or non-degenerative pathological conditions.

### 2.2. Surgical Details

All surgeries were performed by one of three surgeons (clinical experience: >25 years for C.-S.L., 12 years for S.-J.P., and 7 years for J.-S.P.). The correction surgery was performed either through posterior-only surgery using posterior column osteotomy with or without pedicle subtraction osteotomy (PSO), or through a combined anterior–posterior approach using oblique or anterior lumbar interbody fusion. The choice of surgical technique was guided by the patient’s preoperative deformity. While the choice of surgical technique was based on the preoperative deformity status, our institution favored the combined anterior–posterior approach in the later study period.

### 2.3. Clinical Outcome Measurements

At the final follow-up, patient outcomes were assessed using the Oswestry Disability Index (ODI), the Scoliosis Research Society-22r (SRS-22r) questionnaire, and the 36-item Short Form Survey (SF-36) score. In this study, ODI scores were converted to percentage values (%). Poor clinical outcomes were defined by ODI scores > 40 points, as in the previous studies [20,21,22]. Patients were divided into two groups according to their ODI score: group P (poor outcome group, ODI score > 40 points) and group NP (non-poor outcome group, ODI score ≤ 40 points). Although the groups were established based on ODI scores, other HRQOL measures, such as SRS-22r and SF-36 scores, were also compared between the two groups.

### 2.4. Study Variables

To identify the factors affecting poor clinical outcomes, various clinical variables were compared between the P and NP groups for patient factors, surgical factors, radiographic parameters, and mechanical complications.

The patient factors at the index surgery included age, sex, diagnosis (DFB or DLS), American Society of Anesthesiologists (ASA) grade, T-score on bone mineral density (BMD), body mass index (BMI), diabetes mellitus (DM), and smoking status. Surgical factors evaluated in the study included the number of fused segments, the surgical technique used, whether PSO was performed, and the use of pelvic fixation. Whole-spine radiographs in standing posteroanterior and lateral views were taken at three time points: preoperatively, immediate postoperatively (around two weeks post-surgery), and at the final follow-up. These images were assessed to determine radiographic measures such as PI, LL, SS, PT, thoracic kyphosis (TK), T1 pelvic angle (TPA), and the C7SVA. Additionally, in assessing the conventional parameters, the amount of correction was evaluated qualitatively on immediate postoperative radiographs according to the categorical criteria suggested in previous studies [21,23,24,25]. First, postoperative PI-LL mismatch was categorized based on Schwab’s criteria into three groups: under (greater than 10°), matched (within ±10°), and over (less than −10°) [23]. Second, PI-LL was analyzed according to the age-adjusted PI-LL target [21]. The age-adjusted PI-LL target was determined using a previous formula: PI-LL = (age − 55 years)/2 + 3 [25]. Patients were then categorized into three groups based on the difference between their actual PI-LL mismatch and the age-adjusted PI-LL target: under-corrected (offset greater than 10°), matched (offset within ±10°), and over-corrected (offset less than −10°). Additionally, global alignment and proportion (GAP) scores were determined and grouped into three categories: proportioned (P) with scores from 0 to 2, moderately disproportioned (MD) with scores between 3 and 6, and severely disproportioned (SD) with scores of 7 or higher [24].

Regarding mechanical complications, PJK and rod fracture were investigated. In this study, PJK was broadly defined to encompass any kyphotic events at the proximal junction. This included cases where the postoperative proximal junctional angle (PJA) was ≥10°, vertebral fractures at the uppermost instrumented vertebra (UIV) or UIV + 1, failure of UIV fixation, and instances of myelopathy [26]. Rod fracture was defined as discontinuation of the rod at ≥1 site in the construct. The clinical impacts of PJK and rod fracture were further investigated according to the presence of these complications and whether revision surgery was performed.

### 2.5. Statistical Analysis

Categorical variables were presented as frequencies and percentages, while continuous variables were expressed as means with standard deviations. To compare categorical variables between the two groups, Fisher’s exact test was employed, and differences in continuous variables were assessed using Student’s t-test. A multivariate logistic regression analysis was then conducted, incorporating all variables that showed significance in the univariate analysis, to determine independent predictors of poor clinical outcomes. Professional statisticians performed the statistical analyses using SPSS (version 29.0.2.0; IBM Corp., Armonk, NY, USA). A *p*-value of less than 0.05 was regarded as statistically significant.

## 3. Results

Among 320 patients who underwent deformity correction during the study period, 105 patients were included in the final study cohort. The mean follow-up duration was 100.6 ± 32.8 months. The mean age was 66.3 ± 6.8 years at the time of index surgery and 74.7 ± 6.6 years at the final follow-up, and there were 94 women (89.5%). The diagnosis was DFB in 59 patients (56.2%) and DLS in 46 patients (43.8%), and the total number of fused segments was 6.2 levels. Front–back surgery was performed on 56 patients (53.3%), while PSO was carried out in 18 patients (17.1%). At the last follow-up, 52 patients were classified into group P, and 53 into group NP. The mean ODI score in group P was 60.7 points, while 25.7 points in group NP. Other HRQOL measures like both the scores of all individual items and the total sums of SRS-22r and SF-36 scores were significantly better in group NP compared with group P (Table 1).

In terms of the factors that could affect the clinical outcomes, there were no differences in patient factors between the P and NP groups except for the T-score on BMD, which was significantly lower in group P (−1.6 g/cm^2^) than in group NP (−0.8 g/cm^2^) (Table 2).

No notable differences were found in surgical factors, such as the total number of fused segments, use of anterior-posterior surgery, execution of PSO, or pelvic fixation. Similarly, no differences were observed between the two groups in preoperative radiographic factors (Table 3).

Among immediate postoperative radiographic parameters, only the correction amounts relative to the age-adjusted PI-LL target significantly differed between the two groups (Table 3). A relatively higher percentage of patients with overcorrection in group P (19.2%) compared with group NP (5.7%). In contrast, the percentage of patients who achieved matched correction was significantly greater in group NP (71.7%) than in group P (48.1%). There were no differences in terms of Schwab’s PI-LL mismatch criteria or GAP score (Table 3).

**Figure 1:**
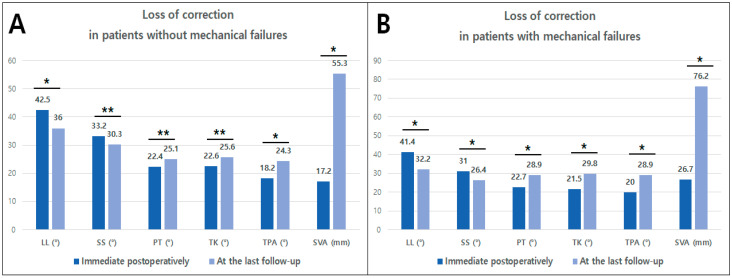
Changes in sagittal parameters between immediate postoperative and at the last follow-up among patients with (**A**) and without (**B**) mechanical failures. * Means *p* < 0.001 and ** means *p* < 0.05. LL indicates lumbar lordosis; SS, sacral slope; PT, pelvic tilt; TK, thoracic kyphosis; TPA, T1 pelvic angle; SVA, sagittal vertical axis.

At the last follow-up, TPA was the only parameter that showed a significant difference between the two groups (30.0° in group P vs. 24.6° in group NP, P = 0.007). Both PT and C7SVA tended to be higher in group P than in group NP, but these differences did not reach statistical significance. Regarding mechanical complications, PJK development significantly affected the clinical outcomes, while the development of rod fracture did not regardless of revision surgery. There were significantly more patients with PJK development in group P than in group NP (53.8% vs. 30.2%). Revision surgery for PJK was performed in significantly more patients in group P than in group NP (23.1% vs. 3.8%) (Table 3). From immediately postoperative to the last follow-up, there were significant decreases in LL and SS, along with significant increases in PT, TK, TPA, and SVA. A similar pattern of correction loss was observed in both patient groups, regardless of whether mechanical failures occurred (Figure 1A,B).

Multivariate analysis identified PJK development as the sole independent risk factor associated with poor clinical outcomes, with an odds ratio (OR) of 3.957 for PJK development and 21.141 for revision surgery related to PJK (Table 4).

**Figure 2:**
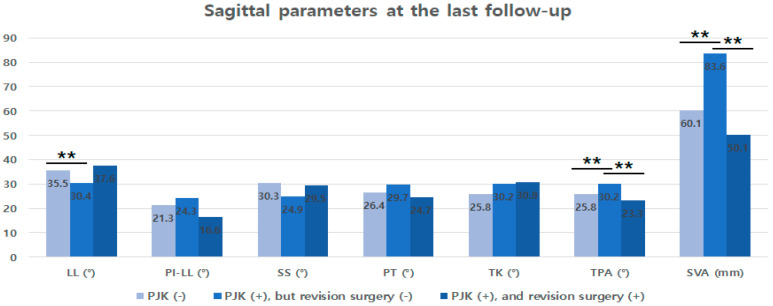
Sagittal parameters at the last follow-up according to PJK development and related revision surgery. Note that TPA and SVA values at the last follow-up were significantly greater in patients with PJK development but were not different between patients without PJK and those who underwent revision surgery for PJK. ** means *p* < 0.05.

Other factors such as T-score, correction amount according to age-adjusted PI-LL, and TPA at the final follow-up, were not significant in multivariate analysis. However, both TPA and SVA values at the last follow-up were significantly greater in patients who developed PJK compared with those who did not. There was no significant difference in these values between patients without PJK and those who underwent revision surgery for PJK (Figure 2).

At the last follow-up, HRQOL measures, such as ODI, several items of SRS-22r, and SF-36 scores, showed a trend of worse outcomes in patients with PJK development and revision surgery followed by patients with PJK but did not undergo revision surgery, and finally, patients without PJK development (Table 5).

## 4. Discussion

In assessing ASD surgery success, pertinent restoration of the optimal spinopelvic alignment has received the most attention. Numerous studies have shown a strong correlation between radiographic alignment and pain or disability in patients with ASD [3,23,27]. However, most of these previous studies were based on 2-year follow-up data. Few studies have reported the predictive factors affecting clinical outcomes over the long-term follow-up period. It is important to ensure good clinical outcomes even during long-term follow-up following any surgical treatment for spinal diseases because as the follow-up period increases, various adverse events can happen, such as loss of correction or mechanical complications, after ASD surgery. Therefore, we believe it is necessary to include such probable adverse events as well as postoperative spinopelvic alignment when evaluating the long-term clinical outcome.

To the best of our knowledge, no previous research has examined long-term clinical outcomes following ASD surgery by combining demographic and radiographic data, and mechanical complications. In this study, we used ODI scores to define the poor outcome group because ODI is a widely recognized tool for assessing disability levels [20,21,28]. However, we also found that other HRQOL measures such as SRS-22r, and SF-36, were significantly worse in group P compared with group NP. Therefore, we believe that the two groups based on ODI scores well represent the poor and non-poor outcome groups.

In the current study, multivariate analysis revealed that the most important factor that led to poor clinical outcomes was PJK development. It is quite understandable that patients with PJK would have inferior clinical outcomes to those of patients without PJK because numerous studies have documented clinical deterioration following PJK development. Kim et al. have reported that pain was prevalent in 0.9% of patients without PJK development compared with 29.4% of patients with PJK, resulting in a lower composite SRS-22r pain score (mean change +12 vs. +0.8) [29]. Bridwell et al. proposed the threshold of PJA of 20° to define the symptomatic PJK. They observed that changes in SRS-22r score were lower in PJK patients, although not significantly different from those in the non-PJK group [30].

At this point, it is notable that PJK development outweighs other factors such as T-score, correction amount relative to the age-adjusted PI-LL, and last TPA, which were only significant in univariate analyses. Although these values were not significant in our multivariate analysis, they are linked to PJK development. Low bone density is a strong risk factor for PJK development, especially bony-failure type PJK [31]. The concept of age-adjusted PI-LL, first introduced by Lafage et al. in 2016 [21], has been regarded as important in PJK prevention. Several studies have demonstrated that aligning with the age-adjusted PI-LL target significantly lowers the risk of developing PJK [32,33]. Recently, Park et al. demonstrated that overcorrection relative to the age-adjusted PI-LL target is associated with a higher risk of PJK and poor clinical outcomes [34]. In this study, we observed a significantly higher number of patients in group P who had overcorrection compared with the age-adjusted PI-LL target, in contrast to those in group NP (Table 3). We infer from this result that overcorrection relative to the age-adjusted PI-LL target correlates with poor clinical outcomes by increasing the risk of PJK.

**Figure 3:**
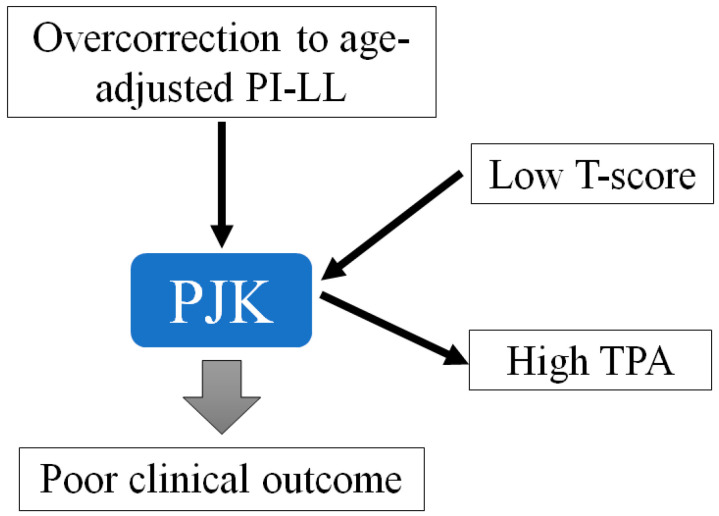
Diagram showing the factors affecting the PJK and clinical outcomes. PI indicates pelvic incidence; LL, lumbar lordosis; PJK, proximal junctional kyphosis; TPA, T1 pelvic angle.

Finally, TPA at the last follow-up was significantly greater in group P than in group NP. It is well-known that a higher TPA is associated with poorer clinical outcomes [22,28]. The increased TPA at the last follow-up compared with immediate postoperatively might result from correction loss during the follow-up period, as we documented a significant loss of correction over time (Figure 1A,B). However, a high TPA could also be the result of PJK development. Once the PJK occurs, C7SVA tends to increase, and the pelvis rotates backward to compensate for the forward posture. Due to these coupled mechanisms, TPA would increase after PJK development, as shown in Figure 2. Although a low T-score on BMD, overcorrection relative to the age-adjusted PI-LL at immediate postoperative and the final high TPA were not significant in the multivariate analysis, and we believe that all these factors are related to PJK development (Figure 3).

Therefore, we suggest that the active management of poor bone quality before and after surgery, combined with proper alignment restoration according to the age-adjusted PI-LL target during surgery, is crucial for preventing PJK and achieving favorable long-term clinical outcomes.

Lastly, we found that patients who underwent revision surgery for PJK had worse clinical outcomes compared with patients with PJK but without revision surgery, despite significant improvement in critical radiographic parameters like TPA and SVA after the revision surgery (Figure 2). This might be because a few patients in this study underwent revision surgery due to spinal cord compression. In a recent study, Ha et al. reported that the prognosis of neurologic deficit after revision surgery for PJK with neurologic involvement is not favorable, with over 50% of patients experiencing no improvement in their neurologic status [35].

A few limitations should be acknowledged in this study. First, the number of enrolled patients was relatively small compared with recent ASD studies. However, this might be attributed to our strict inclusion and exclusion criteria regarding radiographic and clinical data, as well as the long follow-up duration. Second, although we demonstrated that PJK development is most responsible for poor clinical outcomes during long-term follow-up, there might be a few patients who did not experience clinical deterioration despite PJK development. Lastly, our study is limited by its single-center design and the lack of diversity in patient populations. Future multicenter studies are necessary to enhance the generalizability and cohort size.

## 5. Conclusions

This study identified PJK development as the sole independent factor contributing to poor clinical outcomes during long-term follow-up. In contrast, rod fractures were not significantly associated with poor outcomes, even in cases requiring revision surgery. While low T-scores, overcorrection relative to the age-adjusted PI-LL, and high TPAs at the last follow-up were linked to poor outcomes in univariate analysis, these factors are closely related to PJK development. Thus, preventing PJK is crucial for ensuring long-term success following ASD surgery.

## Figures and Tables

**Table 1:** Comparison of the last-follow-up HRQOLs according to the two groups.

		Group P	Group NP	*p* *
**ODI**		60.7 ± 13.8	25.7 ± 11.4	**<0.001**
SRS-22r	Function	2.3 ± 0.6	3.4 ± 0.8	**<0.001**
	Pain	2.6 ± 0.9	4.0 ± 0.5	**<0.001**
	Appearance	2.3 ± 0.6	3.5 ± 0.7	**<0.001**
	Mental health	2.3 ± 0.7	3.7 ± 0.8	**<0.001**
	Satisfaction	2.9 ± 0.7	3.9 ± 0.7	**<0.001**
	Total	2.4 ± 0.5	3.7 ± 0.6	**<0.001**
SF-36	Physical functioning	18.2 ± 17.1	51.3 ± 25.2	**<0.001**
	Role—physical	40.0 ± 25.9	64.3 ± 29.7	**<0.001**
	Bodily pain	39.6 ± 21.5	63.2 ± 20.5	**<0.001**
	General Health	26.4 ± 15.6	50.9 ± 22.2	**<0.001**
	Vitality	34.2 ± 18.6	51.4 ± 21.5	**<0.001**
	Social functioning	36.6 ± 26.9	79.0 ± 21.3	**<0.001**
	Role—emotional	40.4 ± 20.1	66.8 ± 25.0	**0.002**
	Mental health	41.3 ± 20.1	66.8 ± 25.0	**<0.001**
	Physical component summary	31.1 ± 15.1	57.4 ± 18.3	**<0.001**
	Mental component summary	37.8 ± 18.4	67.9 ± 20.4	**<0.001**

Data are presented as the mean ± SD. * Bold *p* values mean statistical significance. HRQOL indicates health-related quality of life; Group P, poor clinical outcome group; Group NP, non-poor clinical outcome group; ODI, Oswestry disability index; SRS-22r, Scoliosis Research Society-22r; SF-36, 36-item short-form health survey.

**Table 2:** Comparison of patient’s and surgical factors according to the two groups.

		Group P	Group NP	*p* *
Patient factors				
	Age at the index surgery (yr)	66.8 ± 7.3	65.9 ± 6.3	0.473
	Age at the last follow-up (yr)	75.5 ± 6.6	73.9 ± 6.6	0.212
	Female:male, *n* (%)	48:4 (92.3%:7.7%)	46:7 (86.8%:13.2%)	0.526
	DFB:DLS, *n* (%)	31:21 (59.6%:40.4%)	28:25 (52.8%:47.2%)	0.557
	ASA grade	2.0 ± 0.4	1.9 ± 0.5	0.552
	T-score on BMD (g/cm^2^)	−1.6 ± 1.7	−0.8 ± 1.7	**0.024**
	BMI (kg/m^2^)	26.2 ± 3.7	25.4 ± 3.6	0.244
	DM, *n* (%)	13 (25.0%)	6 (11.3%)	0.081
	Smoking status, *n* (%)	6 (11.5%)	3 (5.7%)	0.067
Surgical factors				
	No. of total segments fused	6.3 ± 2.2	6.1 ± 2.7	0.689
	Front–back surgery, *n* (%)	23 (44.2%)	26 (49.1%)	0.697
	Application of PSO, *n* (%)	12 (23.1%)	6 (11.3%)	0.127
	Pelvic fixation, *n* (%)	29 (55.8%)	30 (56.6%)	1.000

Data are presented as the mean ± SD or as the number of patients (percentage). * Bold *p* values mean statistical significance. Group P indicates poor clinical outcome group; Group NP, non-poor clinical outcome group; DFB, degenerative flatback; DLKS, degenerative lumbar scoliosis; ASA, American Society of Anesthesiologists; BMD, bone mineral density; BMI, body mass index; DM, diabetes mellitus; PSO, pedicle subtraction osteotomy.

**Table 3:** Comparison of radiographic factors and mechanical failure according to the two groups.

		Group P	Group NP	*p* *
Preoperatively				
	PI (°)	55.1 ± 10.6	53.8 ± 10.5	0.516
	LL (°)	16.1 ± 21.0	21.1 ± 19.7	0.209
	PI-LL (°)	39.1 ± 21.7	32.7 ± 16.9	0.096
	SS (°)	22.7 ± 11.2	23.8 ± 11.0	0.616
	PT (°)	32.4 ± 12.2	30.0 ± 8.5	0.239
	TK (°)	11.7 ± 14.4	16.2 ± 15.4	0.127
	TPA (°)	32.3 ± 11.9	28.8 ± 9.9	0.113
	C7SVA (mm)	81.1 ± 53.3	66.7 ± 49.4	0.153
Immediate postoperatively				
	LL (°)	41.9 ± 11.2	41.8 ± 10.1	0.953
	PI-LL (°)	13.0 ± 12.9	12.1 ± 9.6	0.684
	SS (°)	31.4 ± 7.9	32.1 ± 8.9	0.668
	PT (°)	23.2 ± 8.9	21.9 ± 8.4	0.428
	TK (°)	21.1 ± 10.1	22.7 ± 10.5	0.438
	TPA (°)	20.7 ± 8.8	18.1 ± 27.9	0.091
	C7SVA (mm)	28.2 ± 31.3	18.4 ± 27.8	0.092
	Grouping by Schwab’s criteria			0.559
	Under (PI-LL mismatch > 10°), *n* (%)	30 (57.7%)	27 (50.9%)	
	Matched (PI-LL mismatch ≤ ±10°), *n* (%)	22 (42.3%)	26 (49.1%)	
	Over (PI-LL mismatch < −10°), *n* (%)	0	0	
	Grouping by age-adjusted PI-LL target ^†^			**0.026**
	Under (PI-LL offset > 10°), *n* (%)	17 (32.7%)	12 (22.6%)	
	Matched (PI-LL offset ≤ ±10°), *n* (%)	25 (48.1%)	38 (71.7%)	
	Over (PI-LL offset < −10°), *n* (%)	10 (19.2%)	3 (5.7%)	
	Grouping by GAP score ^‡^			0.632
	Proportioned, *n* (%)	11 (21.2%)	9 (17.0%)	
	Moderately disproportioned, *n* (%)	17 (32.7%)	22 (41.5%)	
	Severely disproportioned, *n* (%)	24 (46.2%)	22 (41.5%)	
At the last follow-up				
	LL (°)	33.9 ± 12.9	33.3 ± 11.7	0.799
	PI-LL (°)	23.2 ± 17.9	20.6 ± 11.9	0.378
	SS (°)	27.9 ± 8.2	27.9 ± 8.7	0.983
	PT (°)	29.2 ± 10.4	25.9 ± 8.7	0.087
	TK (°)	29.3 ± 14.8	27.3 ± 13.7	0.491
	TPA (°)	30.0 ± 11.9	24.6 ± 7.6	**0.007**
	C7SVA (mm)	77.0 ± 53.0	60.0 ± 42.2	0.077
Mechanical complications (PJK)				**<0.001**
	No PJK, *n* (%)	12 (23.1%)	35 (66.0%)	
	PJK, but no revision surgery, *n* (%)	28 (53.8%)	16 (30.2%)	
	Revision surgery for PJK, *n* (%)	12 (23.1%)	2 (3.8%)	
Mechanical complications (Rod fracture)				0.792
	No rod fracture, *n* (%)	41 (78.8%)	43 (81.1%)	
	Rod fracture, but no revision surgery, *n* (%)	8 (15.4%)	6 (11.3%)	
	Revision surgery for rod fracture, *n* (%)	3 (5.8%)	4 (7.5%)	

Data are presented as the mean ± SD or as the number of patients (percentage). * Bold *p* values mean statistical significance. ^†^ Age-adjusted PI-LL target was calculated as follows: Age-adjusted PI-LL target = (Age − 55)/2 + 3. Offset was calculated as the following formula: (actual PI-LL) − (age-adjusted PI-LL target). According to offset, Under means offset > 10°, Matched means offset within ±10°, and Over means offset < −10°. ^‡^ “Proportioned” in GAP score means a total score of 0–2, “moderately disproportioned” 3–6, and “severely disproportioned” ≥ 7. Group P indicates a poor clinical outcome group; Group NP, a non-poor clinical outcome group; PI, pelvic incidence; LL, lumbar lordosis; SS, sacral slope; PT, pelvic tilt; TPA, T1 pelvic angle; C7SVA, C7 sagittal vertical axis; GAP, global alignment and proportion; PJK, proximal junctional kyphosis.

**Table 4:** Multivariate logistic regression analysis of risk factors to cause poor clinical outcome.

Variables	B	S.E	Wald	*p* *	Exp (B) (95% CI)
T-score on BMD (g/cm^2^)	−0.141	0.156	0.817	0.366	0.868 (0.639–1.180)
Categories by age-adjusted PI-LL target ^†^			3.304	0.192	
Matched (vs. Under)	−0.372	0.641	0.336	0.562	0.690 (0.196–2.423)
Over (vs. Under)	1.053	1.024	1.056	0.304	2.865 (0.385–21.325)
Last TPA	0.058	0.032	3.189	0.074	1.060 (0.994–1.129)
Presence of PJK			14.918	**0.001**	
PJK (vs. no PJK)	1.375	0.500	7.570	**0.006**	3.957 (1.485–10.540)
Revision surgery for PJK (vs. no PJK)	3.051	0.897	11.559	**0.001**	21.141 (3.641–122–754)

Stepwise multivariate analysis was performed using variables that had a significance of <0.05 in the univariate analyses. B means regression coefficient; S.E, standard error. * Bold *p*-values indicate statistical significance. ^†^ Age-adjusted PI-LL target was calculated as follows: Age-adjusted PI-LL target = (Age − 55)/2 + 3. Offset was calculated as the following formula: (actual PI-LL) − (age-adjusted PI-LL target). According to offset, Under means offset > 10°, Matched means offset within ±10°, and Over means offset < −10°. BMD indicates bone mineral density; PI, pelvic incidence; LL, lumbar lordosis; TPA, T1 pelvic angle; PJK, proximal junctional kyphosis.

**Table 5:** The last-follow-up HRQOLs according to PJK development.

		PJK (−) (*N* = 47)	PJK (+), Revision (−) (*N* = 44)	PJK (+), Revision (+) (*N* = 14)	*p* * (ANOVA)
ODI		34.9 ± 21.0	47.6 ± 18.5	56.4 ± 23.5	**0.001**
SRS-22r	Function	3.4 ± 0.8	2.6 ± 0.7	2.6 ± 0.7	**<0.001**
	Pain	3.9 ± 0.8	3.2 ± 0.9	2.7 ± 0.9	**0.004**
	Appearance	3.5 ± 0.8	2.6 ± 0.8	2.8 ± 0.8	**0.001**
	Mental health	3.7 ± 0.9	2.7 ± 1.0	2.7 ± 0.5	**<0.001**
	Satisfaction	3.7 ± 0.8	3.5 ± 0.8	2.9 ± 0.9	0.054
	Total	3.6 ± 0.7	2.9 ± 0.7	2.7 ± 0.6	**0.001**
SF-36	Physical functioning	46.3 ± 28.6	29.4 ± 24.7	25.0 ± 24.0	**0.049**
	Role—physical	62.8 ± 32.6	47.7 ± 29.1	41.7 ± 22.7	0.124
	Bodily pain	59.0 ± 23.4	44.0 ± 23.8	56.5 ± 20.9	0.081
	General Health	49.3 ± 20.5	32.9 ± 23.7	32.2 ± 16.6	**0.030**
	Vitality	47.8 ± 22.6	38.9 ± 22.2	43.1 ± 18.1	0.392
	Social functioning	72.5 ± 29.9	51.4 ± 32.8	44.4 ± 25.1	**0.031**
	Role—emotional	65.8 ± 36.7	56.4 ± 34.5	38.9 ± 17.7	0.142
	Mental health	60.3 ± 27.3	50.2 ± 27.6	51.7 ± 14.6	0.410
	Physical component summary	54.3 ± 21.7	38.5 ± 21.2	38.8 ± 11.4	**0.027**
	Mental component summary	61.1 ± 25.3	49.5 ± 26.1	52.8 ± 24.6	0.151

Data are presented as the mean ± SD. (+) indicates that the event has occurred; (−) indicates that the event has not occurred. * Bold *p* values mean statistical significance. HRQOL indicates health-related quality of life; PJK, proximal junctional kyphosis; ODI, Oswestry disability index; SRS-22r, Scoliosis Research Society-22r; SF-36, 36-item short-form health survey.

## Data Availability

Data used in this study can be shared upon reasonable request from the journal. However, it can be limited due to patient privacy and ethical restrictions.
